# Characterization of Dense Granule Metalloproteinase INS-16 in *Cryptosporidium parvum*

**DOI:** 10.3390/ijms23147617

**Published:** 2022-07-10

**Authors:** Hao Cui, Rui Xu, Yu Li, Yaqiong Guo, Ziding Zhang, Lihua Xiao, Yaoyu Feng, Na Li

**Affiliations:** 1 Guangdong Laboratory for Lingnan Modern Agriculture, Center for Emerging and Zoonotic Diseases, College of Veterinary Medicine, South China Agricultural University, Guangzhou 510642, China; m13422049524@163.com (H.C.); piaooluo@gmail.com (R.X.); guoyq@scau.edu.cn (Y.G.); lxiao1961@gmail.com (L.X.); 2 State Key Laboratory of Agrobiotechnology, College of Biological Sciences, China Agricultural University, Beijing 100083, China; yu_li_protein@cau.edu.cn (Y.L.); zidingzhang@cau.edu.cn (Z.Z.)

**Keywords:** *Cryptosporidium parvum*, metalloproteinase, expression differences, invasion

## Abstract

The protozoan pathogen *Cryptosporidium parvum* infects intestinal epithelial cells and causes diarrhea in humans and young animals. Among the more than 20 genes encoding insulinase-like metalloproteinases (INS), two are paralogs with high sequence identity. In this study, one of them, INS-16 encoded by the cgd3_4270 gene, was expressed and characterized in a comparative study of its sibling, INS-15 encoded by the cgd3_4260 gene. A full-length INS-16 protein and its active domain I were expressed in *Escherichia coli*, and antibodies against the domain I and an INS-16-specific peptide were produced in rabbits. In the analysis of the crude extract of oocysts, a ~60 kDa fragment of INS-16 rather than the full protein was recognized by polyclonal antibodies against the specific peptide, indicating that INS-16 undergoes proteolytic cleavage before maturation. The expression of the *ins-16* gene peaked at the invasion phase of in vitro *C. parvum* culture, with the documented expression of the protein in both sporozoites and merozoites. Localization studies with antibodies showed significant differences in the distribution of the native INS-15 and INS-16 proteins in sporozoites and merozoites. INS-16 was identified as a dense granule protein in sporozoites and macrogamonts but was mostly expressed at the apical end of merozoites. We screened 48 candidate INS-16 inhibitors from the molecular docking of INS-16. Among them, two inhibited the growth of *C. parvum* in vitro (EC_50_ = 1.058 µM and 2.089 µM). The results of this study suggest that INS-16 may have important roles in the development of *C. parvum* and could be a valid target for the development of effective treatments.

## 1. Introduction

*Cryptosporidium* spp. are gastrointestinal pathogens that can cause severe diarrhea in humans and various animals [1]. Young children in developing countries infected with *Cryptosporidium* spp. can develop malnutrition and cognitive impairments in addition to clinical illness [2]. In industrialized countries, waterborne outbreaks of cryptosporidiosis are common [3]. Among the more than 40 named *Cryptosporidium* species, *Cryptosporidium parvum* is the main species for cryptosporidiosis in farm animals and one of the two dominant species in humans [4]. Currently, there is a lack of effective drugs against *Cryptosporidium* spp. Although significant progress has been made in the development of novel drugs against cryptosporidiosis in recent years, we still have poor understanding of the biology of the pathogens [5].

The invasion of host cells by apicomplexan parasites is a complex process mediated by receptors and ligands that involve many proteins on both sides [6]. Secreted proteases and protein kinases in secretory organelles of apicomplexans can modify invasion-related proteins or host cell activities, thus playing important roles in invasion [7]. Comparative genomics analysis of multiple *Cryptosporidium* species has revealed the presence of numerous genes encoding secreted proteases in the compact genome. Among them, insulinase-like metalloproteinases (INS) are one of the largest protease families with 22 members in *C. parvum* [8,9]. As the *Cryptosporidium* genome is only 9 Mb in size and most of the ~4000 genes are single copied, INS probably play important roles in the invasion and development of *Cryptosporidium* spp.

INS are zinc metalloproteinases of the M16 family, which are widely distributed in nature and divided into the M16A, M16B, and M16C subfamilies. The M16 metalloproteinases are characterized by the presence of a functional domain containing an inversion of the thermolysin zinc-binding motif, HXXEH [10]. They have a wide range of substrates and cleave many proteins and small peptides including insulin, β-amyloid, and glucagon [11,12]. In apicomplexans, falcilysin is an M16C insulinase involved in hemoglobin catabolism and may function as two different proteases in two subcellular organelles of *Plasmodium falciparum* after proteolytic processing [13,14]. An M16A protease, toxolysin-1 (TLN1) of *Toxoplasma gondii*, is a rhoptry protein that is released during the invasion of host cells. Both the C-terminus and N-terminus of TLN1 undergo cleavage before the maturation of the protease [15]. Another toxolysin, TLN4, is located in the micronemes and involved in parasite fitness [16].

Transcriptome data from *C. parvum* indicate that some INS genes are highly expressed in the invasion stages of the pathogen [17]. In a recent study, one INS protein of *C. parvum*, INS20-19, was shown to be potentially involved in invasion or early developmental processes [18]. Another *C. parvum* INS protein, INS-15, appeared to be post-translationally processed as several fragments and have biological activities similar to toxolysins [19]. Another INS protein, INS-1, is expressed in secretory vesicles within the pathogen and contributes to the formation of macrogamonts [20]. Nevertheless, the role, processing, and trafficking of other INS remain unclear.

In this study, we characterized INS-16 encoded by the cgd3_4270 gene and examined its expression patterns in developmental stages of *C. parvum*. Two inhibitors of the metalloproteinase were identified through virtual screening, which reduced the growth of the pathogen in vitro. In addition, we compared the expression of INS-16 and INS-15, two metalloproteinases with high sequence identity and encoded by neighboring genes. The results suggest that the two INS are located in different organelles and have different biological functions.

## 2. Results

### 2.1. Characteristics of INS-16 and INS-16 Domain I

**Figure 1:**
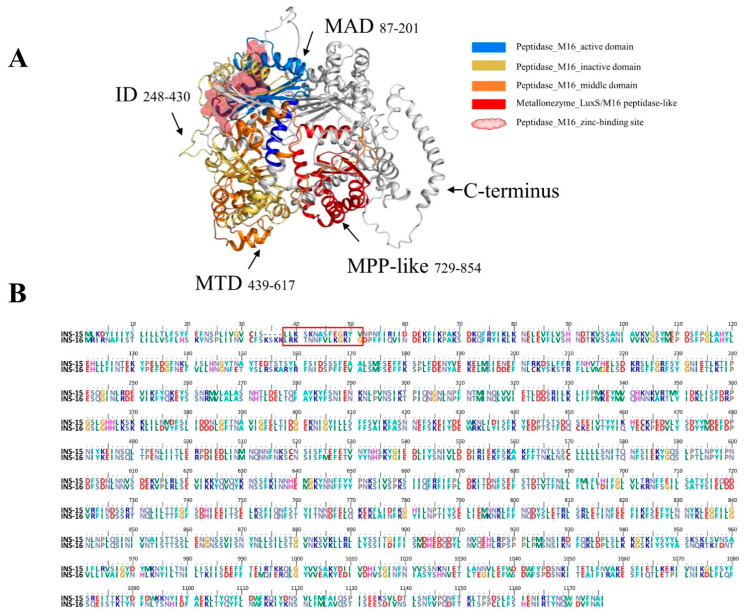
Domain structure and specific amino acid sequence of INS-16 of *Cryptosporidium parvum*. (**A**) Predicted tertiary structures of INS-16. INS-16 have four classic M16 domains; the ribbon model is colored based on the M16 active domain (MAD), inactive domain (ID), the middle domain (MTD), and M16 peptidase-like domain (MPP-like). Black arrows indicate the region of these domains in the model and the amino acid sequence. The red spheroid located between the active domain and the inactive domain indicates the zinc-binding site of INS-16. (**B**) Alignment of amino acid sequences of INS-15 and INS-16. The red box shows the amino acid sequence of INS-16-specific peptide (LRKTNNFVLKGKIG) used in the study.

INS-16 is an M16A secretory metalloproteinase of 1176 amino acids and consists of four classic domains of INS with the key functional motif of HXXEH in the M16 active domain (Figure 1A). It differs from INS-15 in the number of domains, and it has one more M16 peptidase-like domain at the C-terminus than INS-15. Most of the sequence differences between the two INS are in the N- terminus and C-terminus, especially amino acids 1 to 60 (Figure 1B). Using DNA extracted from *C. parvum* oocysts as the template, we successfully cloned the full-length cgd3_4270 gene and its domain I fragment (Figure S1A,D), producing recombinant proteins in *E. coli* (Figure S1B,E). The recombinant proteins were purified using Ni-NTA affinity chromatography, with the purity being confirmed using SDS-PAGE analysis (Figure S1C,F).

### 2.2. Cross-Reactivity between INS-15 and INS-16

**Figure 2:**
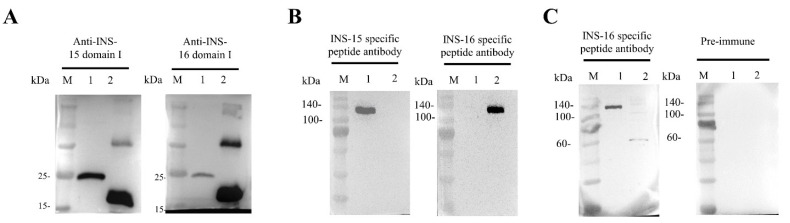
Western blot analysis of cross-reactivity and native protein expression of INS-15 and INS-16. (**A**) Cross-reactivity of antibodies between INS-15 and INS-16. Lane M: protein marker; Lane 1: purified recombinant INS-15 domain I; Lane 2: purified recombinant INS-16 domain I. The image on the left shows the result by using anti-INS-15 domain I as the primary antibodies. The image on the right shows the result by using anti-INS-16 domain I as the primary antibodies. (**B**) Western blot analysis of the specificity of antibodies against INS-16 peptide. Lane M: protein marker; Lane 1: purified recombinant full-length INS-15. Lane 2: purified recombinant full-length INS-16. The picture on the left shows the full-length protein of INS-15 and INS-16 reacting with antibodies against the INS-15 peptide. The picture on the right shows the full-length protein of INS-15 and INS-16 reacting with antibodies against the INS-16 peptide. (**C**) Expression of native INS-16 protein in *C. parvum* sporozoites. Lane M: protein marker; Lane 1: purified recombinant full-length INS-16. Lane 2: *C. parvum* sporozoite lysate. The picture on the right shows the result of proteins reacting with pre-immune serum.

Because INS-16 has high identity to the previously characterized INS-15, we assessed the specificity of the antibodies generated with recombinant INS-16 domain I. In Western blot analysis, the antibodies reacted with the recombinant domain I of both INS-15 and INS-16. Similarly, antibodies against the INS-15 domain I also reacted with the recombinant domain I of both INS-15 (~23 kDa) and INS-16 (~19 kDa) (Figure 2A). However, both antibodies reacted more strongly with their respective proteins. To localize the expression of INS-16, we generated antibodies against the INS-16-specific peptide based on comparisons of the deduced amino acid sequences of INS-15 and INS-16 (Figure 1B). The result of Western blot analysis showed the antibodies against the INS-16-specific peptide only react with the full-length INS-16 protein (~134 kDa). Similarly, the antibodies against the INS-15-specific peptide also only reacted with the full-length INS-15 protein (~137 kDa) (Figure 2B). The antibodies against the two specific peptides did not have any cross-reactivity in Western blot analysis with either INS-15 or INS-16.

### 2.3. Proteolytical Processing of Native INS-16

To assess the expression of the native INS-16 protein in *C. parvum*, we used antibodies generated against the INS-16-specific peptide in the Western blot analysis of sporozoite lysates. The antibodies reacted with a ~60 kDa fragment of the protein instead of the expected 134 kDa full length protein (Figure 2C), indicating that native INS-16 is likely proteolytically processed after its translation.

### 2.4. Differential Expression of INS-15 and INS-16 in Life Cycle Stages of C. parvum

**Figure 3:**
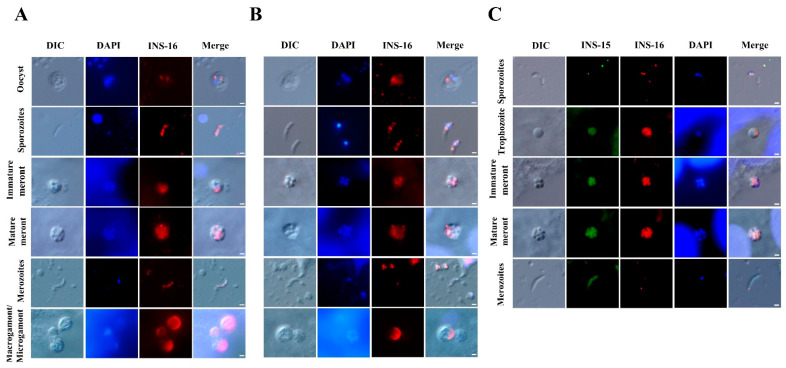
Patterns of INS-16 expression in oocysts, sporozoites, and intracellular stages of *Cryptosporidium parvum* in HCT-8 cells as indicated with immunofluorescence microscopy. (**A**) The localization of INS-16 with antibodies against INS-16 domain I. (**B**) The localization of INS-16 with antibodies against INS-16-specific peptide. The reactivity of the antibodies with oocysts, free sporozoites, and merozoites in infected HCT-8 cells is shown (red). Nuclei were counter-stained with DAPI (blue). Scale bars = 1 μm. (**C**) Co-localization of INS-15 and INS-16 in sporozoite, trophozoite, meront and free merozoite. The reaction of antibodies against INS-15-specific peptide is shown in green fluorescence, while the reaction of antibodies against INS-16-specific peptide is shown in red fluorescence. Nuclei were counter-stained blue with DAPI. Scale bars = 1 μm.

The antibodies against the INS-16 domain I and specific peptide were used to characterize the expression of INS-16 in oocysts, excysted sporozoites, and intracellular stages using immunofluorescence microscopy. The results showed that both antibodies reacted with sporozoites within oocysts, with no significant difference in the staining pattern. However, in excysted sporozoites, anti-INS-16 domain I antibodies reacted with the entire parasites, while antibodies against the INS-16-specific peptide showed a dotty pattern mostly in the middle of the sporozoites in immunofluorescence microscopy. In the immunofluorescence analysis of intracellular developmental stages, both antibodies reacted with meronts with similar staining patterns. The fluorescence signal of antibodies against INS-16 domain I covered almost the entire merozoites, while the fluorescence signal of antibodies against the INS-16-specific peptide was a small dot at the apical end of merozoites. In sexual stage, the antibodies against INS-16 domain I reacted with both the macrogamont and microgamont, while the antibodies against the INS-16-specific peptide reacted mostly with one side of the macrogamont with no reactivity to microgamonts (Figure 3A,B).

To compare the expression between INS-15 and INS-16 in different *C. parvum* life cycle stages, antibodies against INS-15 and INS-16-specific peptides were used in co-localization analysis. The results showed that the signals of INS-15 and INS-16 antibodies in free sporozoites did not overlap completely, with INS-15 being localized in the nucleus of the parasite and INS-16 having more spotty expression over the entire sporozoites, as indicated above. While the staining patterns of INS-15 and INS-16 antibodies were largely similar in trophozoites and meronts, the fluorescence signal of INS-15 antibodies covered almost the entire free merozoites compared with only a small dot near the apical end of free merozoites in the immunofluorescence analysis with INS-16-specific antibodies (Figure 3C).

**Figure 4:**
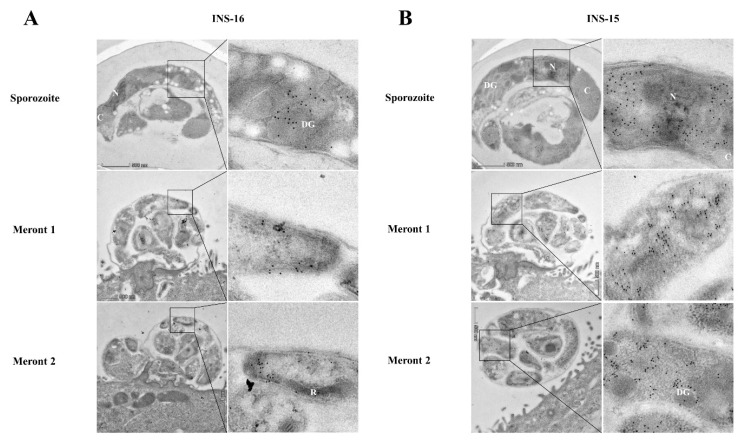
Differences in the distribution of INS-15 and INS-16 expression in organelles of developmental stages of *Cryptosporidium parvum*. The localization of INS-15 and INS-16 in subcellular structures was analyzed using immuno-transmission electron microscopy. The distribution of INS-16 (**A**) and INS-15 (**B**) in sporozoites and meronts. *C. parvum* oocysts and meronts in infected HCT-8 cells were fixed and stained with antibodies against the INS-16 or INS-15-specific peptide followed by 10 nm colloidal gold-conjugated goat anti-rabbit IgG. N, nucleus; DG, dense granule; C, crystalloid body; R, rhoptry. Scale bars, 500 nm.

The expression of INS-16 in sporozoites and merozoites was further examined via immunoelectron microscopy using antibodies against the INS-16-specific peptide. In oocysts, INS-16 expression was mainly in the anterior end and middle of the sporozoites, mostly in dense granules, with some gold particles in the oocyst matrix also (Figure 4A). In contrast, the expression of INS-15 was mainly concentrated around the nucleus of sporozoites, with some gold particles in dense granules (Figure 4B). In meronts of infected HCT-8 cells, INS-16 expression was mainly in the apical end of merozoites, while the expression of INS-15 covered the entire merozoites (Figure 4A,B). This result is consistent with immunofluorescence analysis of INS-15 and INS-16, as indicated above.

### 2.5. Anti-Cryptosporidial Effects of Candidate Inhibitors of INS-16

**Figure 5:**
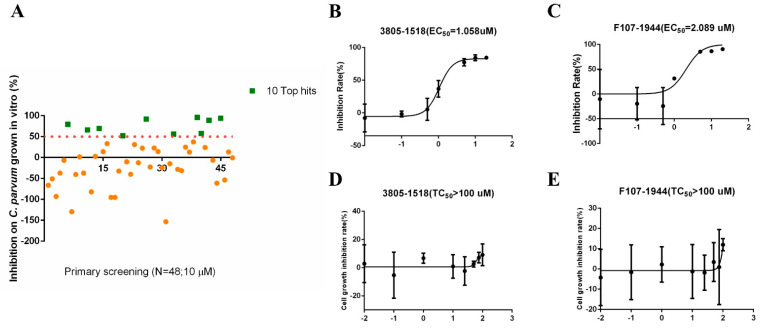
Inhibitory efficacy of candidate INS-16 inhibitors on the development of *Cryptosporidium parvum* in HCT-8 cells. (**A**) Efficacy of all 48 compounds at 10 µM in primary evaluations. Ten compounds with high levels of efficacy (>50%) are marked as green squares. Thirty-eight compounds with low levels of efficacy (<50%) are marked as yellow dots. (**B**,**C**) Dose–response curves of compound 3805-1518 and F107-1944 on *C. parvum* growth. The two compounds can inhibit *C. parvum* growth by 50% at the concentration of 1.058 µM and 2.089 µM, respectively. The anti-cryptosporidial activities were determined by using qRT-PCR. (**D**,**E**) Dose–response curves of compound 3805-1518 and F107-1944 on HCT-8 cell growth. The maximum used on HCT-8 cells without any inhibitory effect above 100 µM for 3805-1518 and F107-1944. The data shown are means ± SD (n ≥ 3) from one representative of at least three independent experiments.

The molecular docking of the simulated structure of INS-16 identified 100 potential inhibitors of the metalloproteinase. Among them, 48 compounds were evaluated for in vitro effects on the invasion and growth of *C. parvum* in HCT-8 cells using a qRT-PCR assay (Table 1). Ten of the compounds showed >50% growth inhibition at the concentration of 10 µM used in the initial evaluation (Figure 5A). The efficacy of these was further assessed in dose–response experiments. Among them, 3805-1518 and F107-1944 had inhibition rates of over 80% at the concentration of 5 µM, with EC_50_ values of 1.058 µM and 2.089 µM, respectively (Figure 5B,C). The two compounds displayed low cytotoxicity on HCT-8 cells, with TC_50_ values > 100 µM (Figure 5D,E).

## 3. Discussion

In this study, a comparative study of INS-16 was conducted together with INS-15 expressed by a paralogous gene. The results obtained indicated that despite the high sequence identity and similar gene expression, the two M16 metalloproteinases appear to be expressed in different organelles of the pathogen, and therefore have different biological functions. Previously, several INS members of *C. parvum* have been characterized, including INS1, INS4, INS5, INS6, INS20-19, INS-21, and INS-23 [18,20,21,22,23]. In agreement with data generated from the present study, they seemingly have diverse expression patterns and biological functions.

INS-16, like insulin-degrading enzyme (IDE) in humans, appears to be a classical zinc metalloproteinase that is proteolytically processed before maturation. Domain analysis shows that INS-16 contains one active domain and three inactive or middle domains. This special structure allows the N-terminal zinc-binding active domain of classical zinc metalloproteinase to be connected to the C-terminal domain, forming a closed proteolytic chamber to exert activity [24,25]. In the Western blot analysis, it was shown that INS-16-specific antibodies recognize a ~60 kDa product in *C. parvum* sporozoites, which is much smaller than the recombinant protein expressed in *E. coli*. This proteolytic processing appears to be common in INS of *C. parvum*. In previous studies, several products of different sizes were observed in the detection of INS4, INS6, INS-15, and INS20-19 in crude extracts of sporozoites despite the fact that some *Cryptosporidium* INS do not have four domains. This was attributed to the presence of several putative SΦX(E/D) cleavage sites in the sequences [18,19,21]. The proteolytic processing of INS-16 is similar to two toxolysins in *Toxoplasma gondii*. The Western blot analysis of native TLN4 showed that TLN4 antibodies principally recognized a ~55 kDa product in tachyzoite lysate, which contained the active domain and the first inactive domain of TLN4 [16]. Another toxolysin, TLN1, was also shown to go through cleavage at the C-terminal, generating a product smaller than the predicted size. It was believed that the cleavage of the C-terminal domain provided additional flexibility for substrate binding, allowing the enzyme to cleave larger substrates [15].

Although related, INS-16 and INS-15 could play different biological functions in the invasion or development of *C. parvum*. These two metalloproteinases have significant sequence homology to each other and are encoded by two neighboring genes. Although both genes had the highest expression at 0–2 h of the in vitro infection, the localizations of the protein expression in sporozoites and merozoites are different between the two metalloproteinases. Members of the INS family are located in different organelles and play different functions, as indicted in comparative studies of INS4 and INS6, and INS-21 and INS-23 [21,23]. In immunoelectron microscopy analysis, the expression of INS-16 was mainly detected in dense granules located in the anterior end and middle of sporozoites and merozoites. In contrast, INS-15 expression in sporozoites was mainly confined to areas around the nucleus, as shown in the present (Figure 4A) and previous studies [19]. INS-15, nevertheless, appears to be a dense granule protein present over the entire merozoites of *C. parvum* (Figure 4B). The only other known dense granule protein of *C. parvum*, CpClec, is expressed in both sporozoites and merozoites and mediates the infection of *C. parvum* through Ca^2+^-dependent binding with sulfated proteoglycans on host intestinal epithelial cells [26]. In other apicomplexan parasites, dense granule proteins are involved in the formation of parasitophorous vacuoles and the modification of the host cell activities [27]. Further studies are needed to elucidate the precise functions of diverse members of the INS family of *C. parvum*.

INS-16 is highly expressed in macrogamonts and may play a role in the sexual life stage of *C. parvum*. Indirect immunofluorescence microscopy showed high INS-16 expression in macrogamonts, and this expression pattern was similar but not identical to the previously reported INS1 expression [20]. INS1 expression is more likely located in small vesicles within macrogamonts, while INS-16 is more likely located in the dense granules within macrogamonts. In addition, the transcriptional activity of the ins-16 gene was high at 72 h of in vitro *C. parvum* infection (Figure S2). Therefore, INS-16 may interact with INS1 or other wall-forming proteins to participate in the development of the sexual stages and oocysts of *C. parvum*.

Inhibitors of INS-16 can effectively inhibit the growth of *C. parvum* in vitro, indicating that INS-16 may be an important drug target. Although we failed to obtain the full INS-16 protein with enzymatic activity, based on the structure of human IDE together with known inhibitors, we were able to simulate the active structure of INS-16, leading to the identification of 48 potential inhibitors from the ChemDiv database through molecular docking. Among them, only 3805-1518 and F107-1944 effectively inhibited the growth of *C. parvum* in vitro without significant cytotoxicity to the host cells. Prior to this, there have been no studies on the inhibitors of *C. parvum* INS. In *Plasmodium falciparum*, it was known that piperazine-based hydroxamic acids kill parasites by blocking falcilysin (FLN). These inhibitors can competitively bind to active and substrate recognition sites in the protease therefore inhibit FLN activity [28,29]. At present, the mechanism of INS-16 in the invasion and development of *C. parvum* is not clear. Further structural and genetic manipulation studies are needed to identify the action mechanism of INS-16 and its candidate inhibitors.

## 4. Materials and Methods

### 4.1. Parasite, Host Cells, and In Vitro Infection

Oocysts of the *C. parvum* IOWA isolates were purchased from Waterborne, Inc. (New Orleans, LA, USA), stored at 4 °C, and used within three months. For infection experiments, oocysts were treated with 0.5% sodium hypochlorite on ice for 10 min and washed three times with PBS via centrifugation at 13,200× *g* for 3 min. The treated oocysts were resuspended with RPMI 1640 culture medium for in vitro infection. HCT-8 cells (ATCC, CCL-244, Chinese Academy of Sciences Shanghai Branch) were seeded into 12-well plates, cultured to ~80% confluence, and inoculated with RPMI 1640 culture medium containing *C. parvum* oocysts. Free sporozoites were obtained from the sodium-hypochlorite-treated oocysts via incubation in PBS (pH 7.4) containing 0.25% trypsin and 0.5% taurodeoxycholic acid at 37 °C for 1 h. Free merozoites were collected from RPMI 1640 culture medium harvested from *C. parvum*-infected HCT-8 culture at 36 h via centrifugation.

### 4.2. Expression of Full-Length INS-16 and INS-16 Domain I

Domains in INS-16 were predicted using InterPro 88.0 (https://www.ebi.ac.uk/interpro/ (accessed on 10 March 2022)) and Pfam 31.0 (https://pfam.xfam.org/ (accessed on 10 March 2022)). *Cryptosporidium* genomic DNA was extracted from oocysts by using the QIAamp DNA MINI kit (Qiagen, Dusseldorf, Germany). The full-length cgd3_4270 gene and its domain I sequence were amplified by using the Phusion High-Fidelity DNA Polymerase (Thermo Fisher Scientific, Waltham, MA, USA). The primers used in the PCR amplification of the full cgd3_4270 gene were 5′-CATGCCATGGAATACAATTCACCACTAATAA-3′ (the *Nco*I restriction site underlined) and 5ʹ-AAATCTCGAGGATCGCATTAAAAACATCC-3ʹ (the *Xho*I restriction site underlined), while those for domain I were 5′-GCGGATCCTATATTAAGTTGAAAAATGAACTTGA-3′ (the *BamH* I restriction site underlined) and 5′-CTCTCGAGAAAACGTGTTGATTTGGAATA-3′ (the *Xho*I restriction site underlined). The PCR amplification of the full cgd3_4270 gene was performed under the following conditions: 95 °C for 30 s; 34 cycles of 95 °C for 10 s, 55 °C for 10 s, and 72 °C for 2 min; and 72 °C for 5 min. The PCR amplification of the domain I fragment was performed under the following conditions: 95 °C for 30 s; 34 cycles of 95 °C for 10 s, 56 °C for 10 s, and 72 °C for 1 min; and 72 °C for 5 min. The PCR products generated were purified using the MiniBEST Agarose Gel DNA Extraction Kit (TaKaRa, Tokyo, Japan), digested with corresponding enzymes and inserted into the expression vector pET28a (Novagen, Madison, WI, USA). The recombinant plasmids were transformed into *E. coli* DH5α cells (Weidi Biotech, Shanghai, China) which were subsequently cultured on Luria–Bertani plates with 50 μg/mL kanamycin for 12 h. The recombinant plasmid with the correct sequence was extracted from positive colonies using the MiniBEST Plasmid Purification Kit (TaKaRa) and transformed into BL21-Codon plus (DE3)-RIPL cells (Weidi Biotech). The protein expression was induced by treating the suspension culture with 0.1 mM isopropyl-β-d-thiogalactopyranoside (IPTG) at 18 °C for 6 h. The recombinant protein was purified using Ni-NTA beads (Thermo Fisher Scientific) and analyzed for purity using SDS-PAGE with Coomassie blue G-250 staining.

### 4.3. Preparation of INS-16 Antibodies

Polyclonal antibodies were prepared through immunizations of rabbits with the recombinant INS-16 domain I and the INS-16-specific peptide (amino acid sequence ^38^LRKTNNFVLKGKIG^51^) by GenScript Ltd. (Nanjing, China). They were purified from the immune sera using protein A Sepharose affinity chromatography (Beyotime, Shanghai, China).

### 4.4. Assessment of the Expression of ins-16 Gene

The relative expression levels of the cgd3_4270 gene during the intracellular development of *C. parvum* in HCT-8 cells were determined using qRT-PCR analysis as described previously [17]. Total RNA was extracted from *C. parvum* cultures using an RNeasy Kit (Qiagen), and cDNA was synthesized from it using the RevertAid First Strand cDNA Synthesis Kit (Thermo Fisher Scientific). The primers of the cgd3_4270 gene used in qPCR were 5′-CGCCAATTCAAAACGGTAAT-3′ and 5′-ATTTCAAATGATGGCCCAAG-3′, while those of Cp18S were 5′-CTCCACCAACTAAGAACGGCC-3′ and 5′-TAGAGATTGGAGGTTGTTCCT-3′. The data presented are the results of three independent experiments performed in duplicate.

### 4.5. Western Blot Analysis of Cross-Reactivity of Antibodies

Western blot analysis was used to assess the cross-reactivity of INS-16 domain I antibodies, using the recombinant INS-15 domain I and antibodies against the INS-15 domain I from the previous study as the control [19]. The recombinant domain I and its native proteins extracted from sporozoites via boiling were separated with 10% SDS-PAGE and transferred onto a polyvinylidene fluoride (PVDF) membrane (Merck Millipore, Billerica, MA, USA) at 300 mA for 80 min. The membrane was blocked at room temperature with 5% nonfat milk-TBST for 1 h and incubated with primary antibodies (against the domain I or specific peptide of INS-15 and INS-16) diluted 1:1000 in blocking buffer (5% nonfat milk in TBST) at 4 °C overnight. After being washed three times with TBST, the membrane was incubated at room temperature with horseradish peroxidase (HRP)-conjugated goat-anti-rabbit antibodies (Yeasen, Shanghai, China) diluted 1:2500 in TBST for 1 h. The membrane was washed three times, treated with High-sig ECL Western Blotting Substrate (Tanon, Shanghai, China), and analyzed using Tanon 5200 (Tanon).

### 4.6. Immunofluorescence Assay

Oocysts or sporozoites resuspended in PBS were dried onto slides and fixed with methanol at room temperature for 15 min. For the intracellular stages of *C. parvum*, infected HCT-8 cells grown on coverslips for 24, 30, and 48 h were washed with PBS and fixed. These slides or coverslips were treated with 0.5% Triton-X for 15 min and blocked for nonspecific binding with 5% BSA for 1 h. After being washed with PBS, the slides or coverslips were incubated sequentially with antibodies against the INS-16 domain I or INS-16-specific peptide and Alexa Fluor 594-conjugated Goat Anti-rabbit IgG (Cell Signaling Technology, Danvers, MA, USA), with the nuclei being counter-stained with 4′6-diamidino-2-phenylindole (DAPI) (Sigma-Aldrich, St. Louis, MO, USA). For co-localization, antibodies against the INS-15 domain I and specific peptide were used in some of the immunofluorescence microscopy analyses.

### 4.7. Immunoelectron Microscopy

Oocysts and free sporozoites were fixed at 4 °C in freshly prepared 4% paraformaldehyde (Leagene, Beijing, China) and 0.1% glutaraldehyde (Leagene) for 60 min. They were processed for immunoelectron microscopy (IEM) as described [23].

### 4.8. Inhibition of C. parvum Invasion and Growth by Candidate INS-16 Inhibitors

A total of 48 compounds were selected from the ChemDiv database through the molecular docking of INS-16 as described previously [30] (Table 1). The anti-cryptosporidial effect of these compounds at the concentration of 10 µM were assessed by using qRT-PCR in 44 h infection assays as described [31]. At least two technical replicates were used in the qRT-PCR analysis of each RNA preparation. In a secondary analysis of selective compounds, various concentrations (from 10 nM to 20 µM) of the compounds were used to treat *C. parvum* cultures. The cytotoxicity of 3805-1518 and F107-1944 on HCT-8 cells were assessed using a 24 h MTS assay (Abcam, Cambridge, MA, USA), with OD490 being determined using a microplate analyzer (BioTek, Winooski, VT, USA). These tests were performed in triplicate.

## 5. Conclusions

In conclusion, the results of the present studies revealed clear differences in the expression and subcellular localizations of two INS members, and while INS-16 is highly expressed in dense granules of sporozoites and in macrogamonts, its expression in merozoites is mostly confined to the apical end, indicating that INS-16 likely exerts multiple functions in the invasion and growth of *C. parvum*. These observations need validation through characterizations of the protein and its function using genetic tools developed recently for *C. parvum*.

## Figures and Tables

**Table 1:** Candidate inhibitors selected based on molecular docking of INS-16 of *Cryptosporidium parvum*.

Name	Docking Score	Molecular Formula	Molecular Weight
Y041-9039	−9.56976	C_23_H_22_F_3_N_3_O_4_	461.434
5492-3909	−9.2222	C_29_H_26_N_2_O_5_	482.527
2516-4540	−9.19194	C_17_H_21_N_5_O_5_	375.39
D271-0061	−9.11034	C_21_H_19_N_3_O_3_S	393.459
S350-0140	−8.84257	C_23_H_20_ClN_3_O_4_	437.876
J100-0222	−8.75284	C_20_H_16_N_4_O_3_	360.366
J106-0147	−8.6609	C_22_H_20_ClN_5_O_2_	421.88
Y041-5161	−8.65563	C_21_H_22_N_4_O_4_S	426.489
D126-0066	−8.61337	C_23_H_25_N_3_O_5_	423.462
G756-0189	−8.58072	C_24_H_22_N_4_O_5_	446.455
7706-0348	−8.57229	C_20_H_21_N_5_O_5_	411.411
3805-1490	−8.52138	C_27_H_21_F_3_N_2_O_2_	462.463
J106-0316	−8.51842	C_21_H_20_N_6_O_2_	388.423
D271-0250	−8.48709	C_20_H_14_F_3_N_3_O_2_S	417.404
J106-0442	−8.48696	C_16_H_15_FN_6_O_2_S	374.393
4340-0243	−8.46525	C_23_H_19_F_2_N_3_O_3_	423.412
3805-1498	−8.45846	C_28_H_26_N_2_O_3_	438.518
D392-0203	−8.45818	C_21_H_18_FN_3_O_4_S	427.449
S350-0509	−8.4509	C_23_H_22_ClN_3_O_4_	439.891
D126-0015	−8.43397	C_22_H_23_N_3_O_5_	409.435
5224-0087	−8.41748	C_17_H_22_N_6_O_4_	374.394
G756-2327	−8.41023	C_22_H_16_ClFN_4_O_3_	438.839
D585-0566	−8.38859	C_14_H_12_FN_5_O_2_S	333.341
Y020-1897	−8.3814	C_20_H_17_Cl_2_N_3_O_2_	402.274
Y040-5718	−8.37768	C_21_H_21_N_3_O_3_	363.41
K915-0695	−8.37702	C_24_H_22_N_2_O_4_	402.442
G756-0218	−8.37549	C_22_H_17_FN_4_O_3_	404.394
J033-0201	−8.36529	C_18_H_15_N_5_O_2_S	365.409
D585-0146	−8.34699	C_15_H_12_N_6_O_3_S	356.359
S350-0527	−8.343	C_23_H_21_N_3_O_6_	435.429
S350-0378	−8.34111	C_22_H_18_ClN_3_O_6_	455.848
D074-0205	−8.33361	C_25_H_29_N_3_O_3_	419.516
D392-0185	−8.33336	C_22_H_19_FN_4_O_4_S	454.474
J023-0481	−8.3308	C_21_H_15_F_3_N_4_O_3_S	460.429
J100-0184	−8.32141	C_18_H_16_N_4_O_4_	352.344
D126-0039	−8.31731	C_24_H_27_N_3_O_5_	437.488
D126-0879	−8.31399	C_26_H_31_N_3_O_5_	465.541
D074-0013	−8.30229	C_28_H_25_N_3_O_4_	467.516
J106-0113	−8.27234	C_18_H_17_N_5_O_2_	335.36
Y020-0362	−8.25097	C_17_H_11_N_3_O_2_S_3_	385.483
D271-0262	−8.24798	C_20_H_14_ClF_2_N_3_O_2_S	433.859
C276-1165	−8.22383	C_22_H_23_NO_3_S	381.488
D392-0159	−8.22361	C_22_H_19_ClN_4_O_4_S	470.929
3805-1518	−8.20851	C_29_H_28_N_2_O_4_	468.544
F107-1944	−8.19206	C_26_H_26_N_2_O_5_	446.495
G365-0266	−8.15557	C_20_H_24_N_4_O_3_	368.43
D392-0230	−8.13482	C_20_H_14_BrF_2_N_3_O_2_S	478.31
8018-4025	−8.10157	C_21_H_21_FN_2_O_5_	400.4

## Data Availability

The original contributions presented in the study are included in the article/supplementary material, and further inquiries can be directed to the corresponding authors.

## Supplementary Materials

The following supporting information can be downloaded at: https://www.mdpi.com/article/10.3390/ijms23147617/s1.
