# Supplementary material for: Characterization of Dense Granule Metalloproteinase INS-16 in Cryptosporidium parvum
Source: Int J Mol Sci. 2022 Jul 10;23(14):7617. doi: 10.3390/ijms23147617 (PMC9315855; doi:10.3390/ijms23147617)
Supplement: Supplementary file 1 [file ijms-23-07617-s001.zip › ijms-1800222-supplementary.pdf]

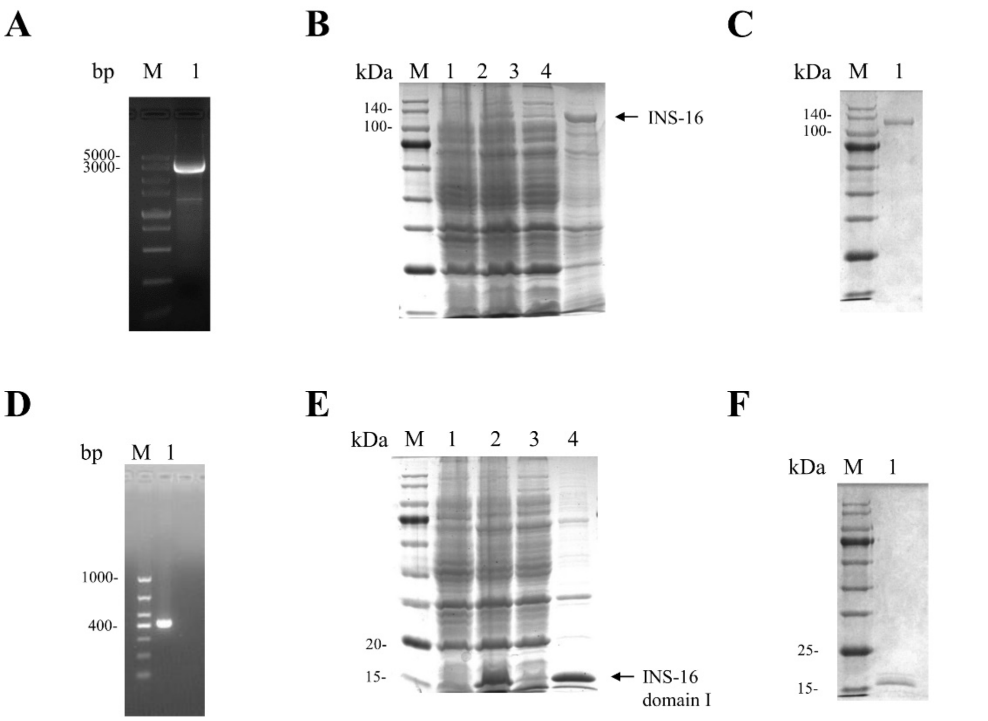

Figure S1. Expression of full-length INS-16 and its domain I of *Cryptosporidium parvum* in *Escherichia coli*. (**A**) PCR amplification of the cgd3_4270 gene. Lane M: DL 5000 DNA marker. Lane 1: cgd3_4270 gene product. (**B**) SDS-PAGE result of INS-16 expression in *E. coli*. Lane M: protein marker. Lane 1: lysate from recombinant bacteria without IPTG induction; Lane 2: lysate from IPTG-induced recombinant bacteria; Lane 3: supernatant of IPTG-induced recombinant bacterial culture; Lane 4: pellet of lysate from IPTG-induced recombinant bacteria. (**C**)INS-16 purification result. Lane M: protein marker. Lane 1: purified INS-16 protein. (**D**) PCR amplification of INS-16 domain I fragment. Lane M: DL 1000 DNA marker. Lane 1: INS-16 domain I fragment amplification product. (**E**) SDS-PAGE result of INS-16 domain I expression in *E. coli*. Lane M: protein marker. Lane 1: lysate from recombinant bacteria without IPTG induction; Lane 2: lysate from IPTG-induced recombinant bacteria; Lane 3: supernatant of IPTG-induced recombinant bacterial culture; Lane 4: pellet of lysate from IPTG-induced recombinant bacteria. (**F**) INS-16 domain I purification result. Lane M: protein marker. Lane 1: purified INS-16 domain I.

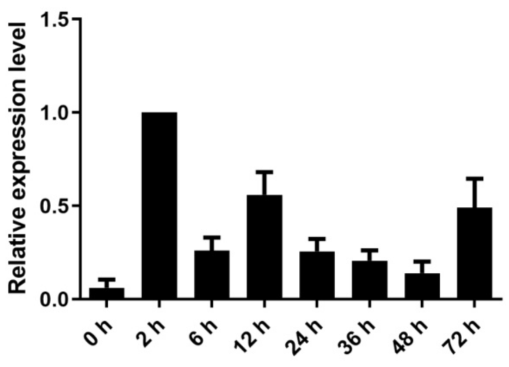

Figure S2. Expression of the cgd3_4270 gene in in vitro culture of *Cryptosporidium parvum*. The relative expression levels of the INS-16 gene during intracellular development of the parasite were examined by qPCR. The data presented are the results of three independent experiments performed in duplicate.
